# The New Empire Pay Hospital

**Published:** 1913-12-20

**Authors:** 


### The New Empire Pay Hospital.

THE BUILDING AND SPECIAL FEATURES.

As we note elsewhere, the Empire Hospital for Paying Patients was opened in Vincent Square this week for inspection prior to the admission of patients. The hospital, which has accommodation at present for forty-four patients and the staff, is built round a central court at the back of the frontage on the square and consists entirely of separate rooms varying in size with the price, which rises from £3 3s. to £10 10s. a week. Care has been taken to avoid bright colouring in the bedrooms, all of which have grey Paripan walls. The furniture is of oak with rounded corners and domes to obviate the collection of dust, and the beds, which are on castors, have moveable heads, which may be lowered if required. Central heating is installed, and Dowsing Heat stoves are provided in the fireplaces. Care has been taken to make the patients' bells easily accessible to them. There are two operating theatres, one for major and one for minor surgery, both of which contain double windows with a hot-water coil for warming purposes between them. The theatre unit also comprises surgeon's dressing-room and sterilising-rooms. Special treatments are given in rooms devoted to them—*e.g.*, *x*-ray room, hydro-electric baths, Nauheim baths, and Dowsing Heat treatment. It is hoped thus to make the institution a centre to which practitioners can resort for special treatments.

The hospital has been built at a cost of over £20,000, and the directors are Lieut.-General Sir James Bevan Edwards, Lord Anglesey, Lady Scarbrough, Mr. Harvey Hilliard, Mr. Benjamin Morgan, and Mr. Herbert Samuelson. The hon. advisory medical committee includes Sir Francis Laking, Sir Victor Horsley, Dr. Arthur Latham, and Mrs. Scharlieb.

